# Tracheal pseudo-tumor caused by herpes simplex virus

**DOI:** 10.1186/2049-6958-8-42

**Published:** 2013-06-25

**Authors:** Stamatis Katsenos, Dimitrios Sampaziotis, Stavros Archondakis

**Affiliations:** 1 Department of Pneumonology, General Army Hospital of Athens, 158 Mesogion & Katehaki Avenue, 115 25 Athens, Greece; 2 Department of Cytopathology, General Army Hospital of Athens, Athens, Greece

**Keywords:** Endotracheal, Herpes simplex virus, Tumor

## Abstract

**Background:**

Herpes simplex virus (HSV) has been shown to cause respiratory tract infections mostly in severely immunocompromised patients. Endobronchial tumor-like lesions have been described very rarely as HSV pulmonary manifestations in critically ill patients or in immunosuppressed individuals.

**Case presentation:**

This case study describes a 75-yr-old male who presented with persistent hoarseness. Fiberoptic bronchoscopy showed marked mucosal thickening protruding in mid and distal trachea causing stenosis. Biopsy specimens demonstrated cytopathological changes consistent with HSV type 1 and 2 infection.

**Conclusions:**

To the best of the authors’ knowledge, this is the first reported case of HSV presenting as an endotracheal tumor in an immunocompetent person.

## Background

Herpes simplex virus (HSV) can cause tracheobronchitis and pneumonitis mostly in severely immunocompromised patients [1,2]. Endobronchial tumor has very rarely been reported as a clinical presentation of HSV in critically ill patients or in individuals with conditions leading to impaired immunity [3,4]. However, this rare clinical manifestation has not been described in normal hosts. Herein, we present the first case, to our knowledge, of endo-tracheal tumor caused by HSV infection in an immunocompetent person.

## Case presentation

A 75-year old male, heavy smoker (70 p/y), was referred to our department for evaluation of persistent hoarseness and associated non-productive cough. He reported the onset of these symptoms two months before his admission. His past medical history included arterial hypertension, hypercholesterolaemia and a resected basal cell skin carcinoma of the neck. He was receiving daily atorvastatin (20 mg), ezetimibe (10 mg) and valsartan plus hydrochlorothiazide (80+12.5 mg). Additionally, he did not mention any immunosuppressive condition or any cytotoxic drug intake.

Chest examination revealed diffuse expiratory wheezing. The rest of physical examination was unremarkable. Initial laboratory testing was normal except for a slightly increased erythrocyte sedimentation rate (35 mm/h) and C-reactive protein (2.5 mg/dl). Standard serum biochemistry tests were within normal range. Spirometry showed decreased FEV_1_, FVC, FEF_25–75_% (57, 63 and 28% of predicted values), increased FEV_1_/FVC ratio (93% of predicted value) and normal FIVI thus representing a moderate restriction with associated small airways obstruction. Arterial blood gases while breathing room air showed mild hypoxemia (PaO_2_: 70 mmHg, PaCO_2_: 37 mmHg, pH: 7.44 and bicarbonate: 27.5 mmol/l). Chest computed tomography showed mid and distal trachea lumen stenosis. No lesion was noted in the lung parenchyma.

**Figure 1:**
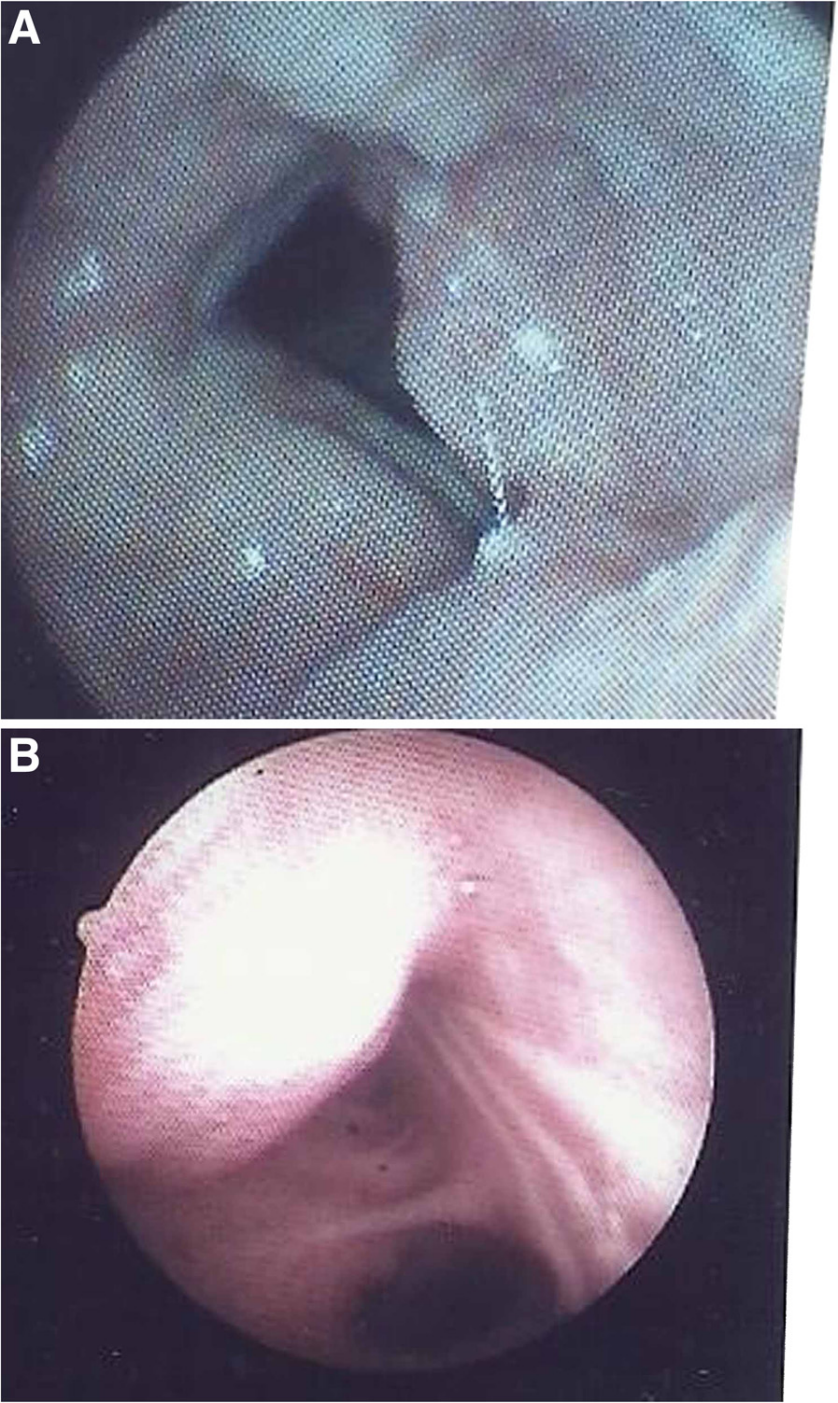
**A Fiberoptic bronchoscopy showing prominent mucosal thickening at the left false and true vocal cord and the anterior commissure of glottis. B**. Irregular and mucosal surfaces protruding in mid and lower third of the trachea.

**Figure 2:**
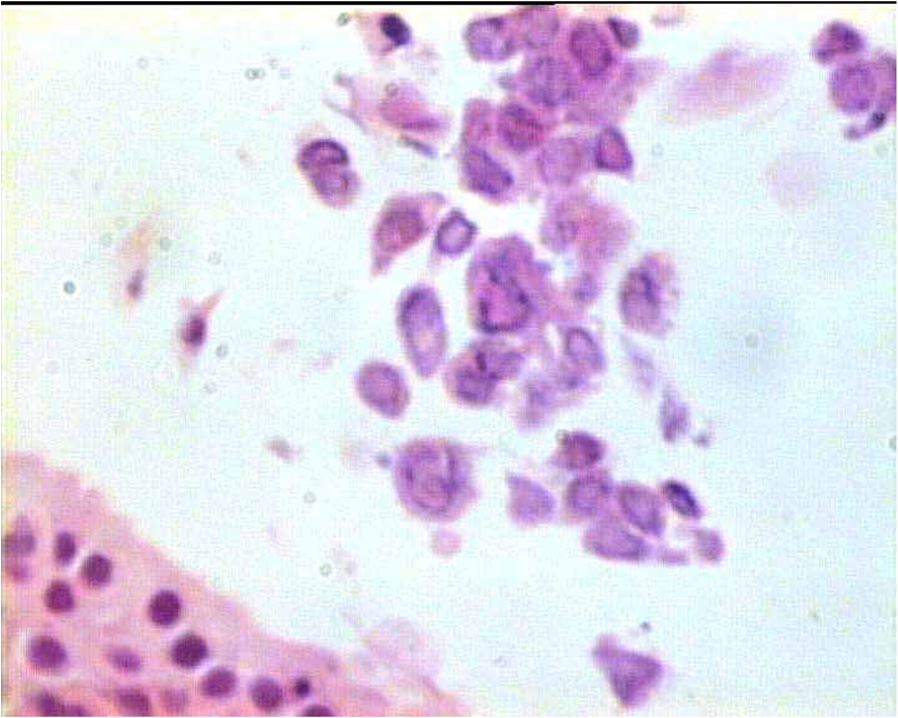
Tracheal biopsy showing intranuclear inclusions (haematoxylin and eosin stain [H&E], original magnification X400).

Fiberoptic bronchoscopy demonstrated prominent mucosal thickening at the left false and true vocal cord and the anterior commissure of glottis (Figure 1A), as well as irregular and mucosal surfaces protruding in mid and lower third of the trachea resulting in lumen stenosis (Figure 1B). Biopsies showed tracheal mucosa fragments that were lined by thickened squamous epithelium with mild hyperplasia and atypia and slight increase of mitotic activity in its basal half. There was no evidence of malignancy. Focally, the epithelial cells demonstrated intranuclear inclusions typical of herpes virus (Figure 2). Similar findings were found in biopsies from lesions of vocal cords. Molecular testing using polymerase chain reaction (PCR) identified HSV type 1 in tissue specimens. Based on histopathology results, the patient received intravenous acyclovir (5 mg/kg every 8 hours) for 7 days followed by oral drug administration for the next two weeks. Three months after the completion of treatment, a repeat fiberoptic bronchoscopy showed substantial regression of the laryngeal and tracheal lesions. No evidence of HSV infection was noted in multiple biopsy specimens. The patient’s voice improved and annual bronchoscopic examination was recommended.

## Discussion

Primary infection with HSV and its reactivation usually occur in immunocompromised patients. Therefore, herpetic tracheobronchitis and pneumonia as pulmonary manifestations have been reported in patients with malignancy, burns, organ transplantation, AIDS or patients receiving corticosteroids [1]. Moreover, herpes tracheobronchitis and bronchopneumonia have been described in immunocompetent patients, particularly in the elderly as well as in non-immunocompromised patients requiring mechanical ventilation [2,3].

Herpes simplex viruses (HSV-1, HSV-2) have been identified as causative agents of lower respiratory tract infections, such as tracheobronchitis and pneumonia. The precise disease mechanism involved is not clearly understood. However, it has been postulated that the virus may reach the lower respiratory tract by aspiration or by contiguous spread after reactivation and shedding in the oropharynx. The presence of macroscopic bronchial lesions, possibly due to local microtrauma and/or pre-existing acute lung injury with distal squamous cell metaplasia, might also have paved the way for distal infection. Thus, local distal reactivation as another mechanism cannot be unequivocally excluded [3].

Although HSV can be cultured from sputum, diagnosis of HSV infection is feasible from the characteristic cytological and histological findings seen in samples obtained by bronchoscopic examination [1]. More specifically, the presence of intranuclear inclusions is considered to be useful cytopathic feature, albeit less sensitive. Furthermore, HSV can be detected by virus culture or quantitatively by nucleic acid amplification techniques. While viral cultures are difficult to handle, PCR has become the most popular diagnostic tool since it is easily performed. In the present case, histological findings as well as molecular testing using PCR were confirmatory of HSV infection.

Only two previous cases of HSV endobronchial masses simulating neoplasm have been reported in immunocompromised patients [4,5]. In particular, the first case describes a HIV-infected patient who presented with ulcerative tracheobronchitis and a cauliflower-like lesion occluding the right upper lobe bronchus. The second case study refers to a patient with severe kyphoscoliosis who was intubated for acute on chronic hypercapnic respiratory failure due to community-acquired pneumonia. Fiberoptic bronchoscopy demonstrated an endobronchial mass in the right middle lobe. Cultures and biopsy specimens demonstrated HSV-1 infection. The endotracheal tumor-like lesions of the patient presented here may have resulted from reactivation of latent infection, because he had HSV-positive serology (IgG without IgM) at the time of diagnosis.

## Conclusion

This is the first reported case, to our knowledge, of HSV type I infection presenting with tracheal tumor-like lesions to generate awareness that this entity may masquerade neoplasm even in the immunocompetent patient.

## Consent

Written informed consent was obtained from the patient for publication of this Case report and any accompanying images. A copy of the written consent is available for review by the Editor-in-Chief of this journal.

## Abbreviations

FEF25-75%: Forced mid-expiratory flow; FEV1: Forced expiratory volume in one second; FIV1: Forced inspiratory volume in one second; FVC: Forced vital capacity; HSV: Herpes simplex virus; PCR: Polymerase chain reaction.

## Competing interest

The authors declare that they have no competing interests.

## Authors’ contributions

SK performed fiberoptic bronchoscopy and endotracheal biopsies, participated in patient follow up and prepared the manuscript. DS and SA carried out the pathology tests and molecular testing. All authors read and approved the final manuscript.
